# Bifurcated optic radiation tractography using diffusion tensor imaging in a patient with traumatic brain injury: A case report

**DOI:** 10.1097/MD.0000000000043746

**Published:** 2025-08-08

**Authors:** Hongsuk Baik, Jongwook Kim, Jeongson Huh, Seansoonsung Hwang, Seyoung Shin, Hyeok Gyu Kwon, MinYoung Kim

**Affiliations:** a Department of Rehabilitation Medicine, CHA Bundang Medical Center, CHA University School of Medicine, Seongnam, Gyeonggi-do, Republic of Korea; b Rehabilitation and Regeneration Research Center, CHA University School of Medicine, Seongnam, Gyeonggi-do, Republic of Korea; c Department of Physical Therapy, College of Health Science, Eulji University, Seongnam, Gyeonggi-do, Republic of Korea.

**Keywords:** case report, diffusion tensor imaging, diffusion tensor tractography, optic radiation, traumatic brain injury

## Abstract

**Rationale:**

Visual field deficits can occur in up to 60% of traumatic brain injury (TBI) cases. The Humphrey visual fields test and visual-evoked potential studies (VEPs) are common diagnostic tools but rely on patient’s participation and can be affected by external factors. Diffusion tensor imaging (DTI) and diffusion tensor tractography (DTT) can provide an objective alternative to evaluating optic radiation (OR) injury. Although previous studies have examined the OR by categorizing it into only left and right tracts, we demonstrate the feasibility of assessing OR injury by subdividing each side into upper and lower regions in a patient with visual field deficits following TBI using DTI and bifurcated DTT.

**Patient concerns:**

In June 2013, a 40-year-old man fell down the stairs, resulting in a large right frontotemporal epidural hemorrhage, a small contusional hemorrhage, and a left frontotemporoparietal subdural hemorrhage. He underwent an emergency decompressive craniectomy. Following the accident, he reported blurred peripheral vision in both eyes. Six years later, he visited the outpatient clinic due to continuous visual impairment.

**Diagnoses:**

He had right upper and left lower quadrantanopsia resulting from OR injury secondary to TBI.

**Interventions:**

DTI data were acquired 6 years after TBI onset.

**Outcomes:**

Four ORs from the patient and 8 healthy controls were reconstructed using DTI and DTT. We set the seed region of interest on the lateral geniculate nucleus, and the target region of interest on the occipital pole along the upper and lower lips of the calcarine fissure, bilaterally. As a result, we divided the ORs into 4 regions; upper right, lower right, upper left, and lower left. The patient’s right upper and left lower ORs showed significantly higher mean diffusivity values and lower tract volume values compared to controls, as well as narrower neural fibers on DTT compared with healthy controls. This finding aligns with the Humphrey visual fields test results, which indicated right upper and left lower quadrantanopsia in the patient.

**Lessons:**

This case demonstrates that DTI and DTT provide an objective assessment of OR injury, independent of patient cooperation. A refined 4-tract OR analysis can allow for more precise assessment of OR injury, potentially enabling the identification of quadrantanopsia.

## 1. Introduction

Visual field deficits (VFD) are common in traumatic brain injury (TBI), affecting up to 60% of patients.^[1]^ Although the Humphrey visual fields test (HVFT), a standard assessment tool for VFD, is straightforward and easy to administer, its result relies on the patient’s ability to actively participate and provide accurate feedback. Visual-evoked potential studies (VEPs) are also used as diagnostic tools, but the results can be influenced by nontarget-related factors, such as the size and contrast of visual stimuli and ability to maintain a steady gaze on the stimuli.^[2]^ Diffusion tensor imaging (DTI) and diffusion tensor tractography (DTT) may be useful alternatives to overcome these limitations by enabling the elective assessment of target neural tracts in the damaged brain tissue, without being affected by the patient’s cooperation or nontarget-related factors.^[3]^ Although several studies have investigated optic radiation (OR) injury in patients with brain injury using DTI and DTT for this reason, previous studies have limited their analysis into 2 broad categories: left and right ORs.^[4]^ This binary approach may overlook the finer anatomical-functional relationships within the visual pathway. In present study, we demonstrate the feasibility of assessing OR injury by subdividing each side into upper and lower regions using DTI and bifurcated DTT analyses in a patient who developed VFD following TBI.

## 2. Case report

A 40-year-old man underwent emergent decompressive craniectomy due to a large right frontotemporal epidural hemorrhage, combined with a small contusional hemorrhage and left frontotemporoparietal subdural hemorrhage, caused by falling down the stairs in June 2013. During recovery from the surgery, a new right centrum semiovale infarction occurred. After the accident, the patient experienced blurred peripheral vision in both eyes. He underwent intensive rehabilitation for 4 months post-onset.

**Figure 1.**
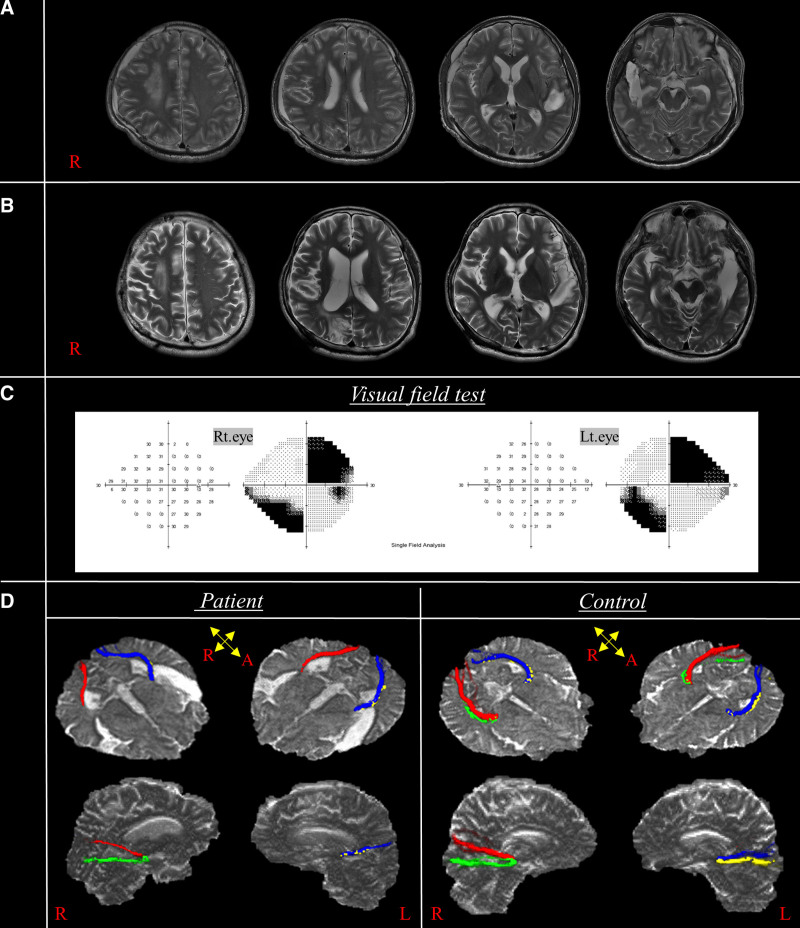
Brain magnetic resonance imaging, diffusion tensor tractography, and visual field test results in the patient. (A) The axial T2 fast spin echo magnetic resonance image taken 2 weeks after injury (June 2013). (B) Axial T2 propeller magnetic resonance image taken 6 years later (July 2019). (C) Automated visual field showing visual field defects in both of the patient’s eyes. (D) Diffusion tensor tractography of the optic radiations in the patient (July 2019) and a control (fiber color: right upper fibers – red, right lower fibers – green, left upper fibers – blue, left lower fibers – yellow).

Six years after discharge, he visited the outpatient clinic due to persistent visual disturbance with a functional status similar to that observed before, including left hemiparesis (Medical Research Council grade 3+ to 4) and intact cognitive function (30 points on the Mini Mental State Examination; Table 1). Brain magnetic resonance imaging (MRI) revealed the known prior infarction in the right centrum semiovale and encephalomalacia (Fig. 1A, B). HVFT indicated right upper and left lower quadrantanopsia in both eyes (Fig. 1C). The quadrant-field VEPs demonstrated no response in the right upper quadrant, and prolonged P100 latency in the left upper quadrant in both eyes, measured at 146 and 160 ms for the left and right eye stimulation, respectively (Table 2).

**Table 1:** Follow-up results of the functional evaluation 6 years after onset.

	2013*	2019
Manual muscle test	Right/left	Right/left
Upper extremity		
Shoulder flexion	Normal/fair	Normal/fair+
Shoulder abduction	Normal/fair	Normal/fair+
Elbow flexion	Normal/fair+	Normal/good−
Elbow extension	Normal/fair+	Normal/good−
Wrist flexion	Normal/fair+	Normal/good−
Wrist extension	Normal/fair+	Normal/good−
Lower extremity		
Hip flexion	Normal/fair+	Normal/good−
Hip extension	Normal/fair+	Normal/fair+
Hip abduction	Normal/fair+	Normal/fair+
Knee extension	Normal/fair+	Normal/good−
Ankle dorsiflexion	Normal/fair	Normal/fair+
Berg balance scale (full 56)	51	55
Trunk impairment scale (full 23)	19	18
Rivermead motility index (full 15)	14	13
Fugl Meyer assessment of the upper extremity (right/left, full 66)	66/57	66/64
Grasp power (right/left, kg)	36/20	40/26
Mini-Mental State Examination (full 30)	30	30

* Functional status at discharge after intensive inpatient rehabilitation.

**Table 2:** P100 latencies in the full field, half field, and quadrant field pattern reversal visual-evoked potential studies (VEPs).

Side (eye)	Stimulation	Recording	P100 latency (ms)
Left	Full-field	Scalp	116
Right	Full-field	Scalp	117
Left	Left half-field	Scalp	119
Right	Left half-field	Scalp	128
Left	Right half-field	Scalp	117
Right	Right half-field	Scalp	119
Left	Left upper quadrant field	Scalp	146*
Right	Left upper quadrant field	Scalp	160*
Left	Right upper quadrant field	Scalp	NR*
Right	Right upper quadrant field	Scalp	NR*
Left	Left lower quadrant field	Scalp	119
Right	Left lower quadrant field	Scalp	121
Left	Right lower quadrant field	Scalp	121
Right	Right lower quadrant field	Scalp	119

VEPs = visual-evoked potential studies.

* Abnormal.

This case report provides written and informed consent from the patient and the study protocol was approved by the Institutional Review Board of CHA Bundang Medical Center (IRB No. 2019-11-057).

### 2.1. Diffusion tensor imaging and tractography

Brain MRI and DTI were performed using a 3T GE Signa System (General Electric, Milwaukee). DTI data were acquired using 2-dimensional axial spin-echo and echo planar imaging with refocusing pulses. The imaging parameters were as follows: acquisition matrix = 128 × 128; field of view = 240 mm × 240 mm; repetition time = 13,000 ms; echo time = 109.1 ms, gradient directions = 25; parallel imaging reduction factor = 2.0; *b* = 1000 s/mm^2^, number of excitations = 2; and slice thickness = 2 mm. All radiological findings were reported by an expert neuroradiologist. Fiber tracking was conducted using probabilistic tractography with default tractography settings in the FMRIB Diffusion Software (Oxford, UK). This included 5000 streamline samples, 0.5 mm step lengths, and a curvature threshold of 0.2. Eddy current correction was subsequently applied to correct the head motion effect and image distortion.

To reconstruct the OR, the seed region of interest was set on the lateral geniculate nucleus, and the target region of interest was set on the occipital pole along the upper and lower lips of the calcarine fissure, bilaterally.^[5,6]^ Values of fractional anisotropy, mean diffusivity (MD), and tract volume (TV) for the OR were measured. To verify the normal range of the above values, 8 age-matched healthy controls (aged between 40 and 64 years) for whom DTI data were obtained under the same conditions were retrospectively selected from our previous DTI study results. DTI parameter values showing more than 2 standard deviations from the normal control values were defined as indicative of an injured OR.

### 2.2. Imaging results

Four ORs for the patient and 8 controls (2 males, 6 females; mean age: 51.3 ± 7.8 years, range: 40–62 years) were reconstructed using DTI and DTT (Fig. 1D). The right upper fiber (Fig. 1D, red) and left lower fiber (Fig. 1D, yellow) of the patient showed decreased fiber volume compared to those of controls. A summary of the DTT parameter values of the patient and controls and the limits of 95% confidence interval calculated from the control group is presented in Table 3. The MD values of the right upper and left lower ORs of the patient were significantly higher than those of the controls (right upper: patient 1.226, control 0.81 [0.72–0.90]; left lower: patient 1.110, control 0.83 [0.73–0.93]), while the TV values of the same tracts were significantly lower than controls (right upper: patient 354, control 1485.9 [431.9–2539.9]; left lower: patient 43, control 1184.6 [732.3–1636.9]). The out-of-reference value in the left lower OR implies a VFD in the right upper quadrant, and that in the right upper OR implies a VFD in the left lower quadrant. In contrast, FA showed no significant difference in the 4 ORs between the patient and controls.

**Table 3:** Diffusion tensor imaging parameters of the optic radiations of patients with traumatic brain injury and normal controls.

			Patient	Controls (range of 2SD)
Fractional anisotropy	Right	Upper	0.35	0.46 (0.32–0.60)
		Lower	0.54	0.47 (0.34–0.60)
	Left	Upper	0.48	0.43 (0.36–0.50)
		Lower	0.49	0.45 (0.35–0.54)
Mean diffusivity	Right	Upper	1.226*	0.81 (0.72–0.90)
		Lower	0.871	0.79 (0.70–0.89)
	Left	Upper	0.853	0.81 (0.72–0.89)
		Lower	1.110*	0.83 (0.73–0.93)
Tract volume	Right	Upper	354*	1485.9 (431.9–2539.9)
		Lower	732	1361 (493.2–2229.3)
	Left	Upper	437	1496 (420.9–2572.9)
		Lower	43*	1184.6 (732.3–1636.9)

* Abnormal.

## 3. Discussion

The patient had right upper and left lower quadrantanopsia following TBI. The history of TBI and previous brain MRI findings suggested the possibility of sequelae of OR injury, potentially caused by ischemia and contusional hemorrhage in the right and left hemispheres, respectively. Using DTI data, the reconstructed tract showed increased MD and decreased TV of the right upper and left lower ORs, which correlated with defective visual field areas.

The MD value increases with vasogenic edema or accumulation of cellular debris from neural injury, while the TV value is determined by the number of neural fibers contained within the neural tract.^[7,8]^ Thus, increments in MD values with decreases in TV values at the 2 ORs indicate past neural tract injury. The DTT results in this case report correlated with the patient’s symptoms and other diagnostic tools, such as HVFT and VEPs.

This case demonstrates the effectiveness of DTI and DTT as tools for objectively evaluating OR injuries that cannot be evaluated by conventional MRI, as they are not affected by patient cooperation or other factors. To the best of our knowledge, this is the first study to analyze OR by subdividing it into 4 distinctive tracts: right upper fibers. right lower fibers, left upper fibers and left lower fibers. This is an advancement on previous studies that were limited to 2 tracts focusing on the left or right ORs. Additionally, this study compares these findings with results obtained from other diagnostic methods. Based on these finding, the study suggests the possibility of identifying quadrantanopsia through a segmented analysis of the OR using DTI and bifurcated DTT analysis. Future studies with larger sample sizes are essential for validating these findings and assessing their wider clinical relevance.

## Author contributions

**Conceptualization:** Hongsuk Baik, Jongwook Kim, MinYoung Kim.

**Data curation:** Hongsuk Baik, Jongwook Kim, Jeongson Huh.

**Methodology:** Hongsuk Baik, Jongwook Kim, Seyoung Shin, Hyeok Gyu Kwon, MinYoung Kim.

**Visualization:** Hongsuk Baik, Jeongson Huh, Seansoonsung Hwang.

**Writing – original draft:** Hongsuk Baik.

**Writing – review & editing:** MinYoung Kim, Seyoung Shin, Hyeok Gyu Kwon.
